# Crystal structure and Hirshfeld surface analysis of 2,2′′′,6,6′′′-tetra­meth­oxy-3,2′:5′,3′′:6′′,3′′′-quaterpyridine

**DOI:** 10.1107/S205698901901274X

**Published:** 2019-09-20

**Authors:** Suk-Hee Moon, Jinho Kim, Ki-Min Park, Youngjin Kang

**Affiliations:** a Department of Food and Nutrition, Kyungnam College of Information and Technology, Busan 47011, Republic of Korea; b Division of Science Education & Department of Chemistry, Kangwon National University, Chuncheon 24341, Republic of Korea; c Research Institute of Natural Science, Gyeongsang National University, Jinju 52828, Republic of Korea

**Keywords:** crystal structure, quaterpyridine derivative, π–π inter­action, C—H⋯π inter­action, Hirshfeld surface analysis

## Abstract

In the title compound, C_24_H_22_N_4_O_4_, the four pyridine rings are tilted slightly with respect to each other. The dihedral angles between the inner and outer pyridine rings are 12.51 (8) and 9.67 (9)°, while that between inner pyridine rings is 20.10 (7)°. Within the mol­ecule, intra­molecular C—H⋯O and C—H⋯N contacts are observed. In the crystal, adjacent mol­ecules are linked by π–π stacking inter­actions between pyridine rings and weak C—H⋯π inter­actions between a methyl H atom and the centroid of a pyridine ring, forming a two-dimensional layer structure extending parallel to the *ac* plane. Hirshfeld surface analysis and two-dimensional fingerprint plots indicate that the most important contributions to the crystal packing are from H⋯H (52.9%) and H⋯C/C⋯H (17.3%) contacts.

## Abstract 2

The title 2,3′-bi­pyridine-based quaterpyridine derivative has a linear geometry. The pyridine rings are tilted slightly with respect to each other. In the crystal, π–π stacking and weak C—H⋯π inter­actions lead to formation of a two-dimensional layer structure.

## Chemical context   

Polypyridines are considered to be strong and versatile chelating ligands for transition-metal ions (Adamski *et al.*, 2014 ▸). This chelating nature provides complexes with diverse architectures possessing unique and useful photophysical properties (Zhong *et al.*, 2013 ▸). Many structural studies of bi- and terpyridine-based metal complexes have been undertaken over the last decades (Kaes *et al.*, 2000 ▸). When bi- or terpyridines are used as building blocks, sophisticated architectures such as helicates and cages can be obtained by self-assembly (Yeung *et al.*, 2011 ▸; Glasson *et al.*, 2008*b* ▸). Although there are number of examples of bi- and terpyridine-based metal complexes with different geometries, structural reports of linear-type quaterpyridines are still scarce (Glasson *et al.*, 2011*b* ▸). Organic compounds bearing 2,3′-bi­pyridine have attracted much inter­est because of their unique properties such as proper coordination modes to late transition-metal ions and high triplet energy. As a result of these characteristics, they are widely used as ligands to develop blue phospho­rescent materials (Zaen *et al.*, 2019 ▸; Lee *et al.*, 2018 ▸). However, no reports of a 2,3′-bi­pyridine-based quaterpyridine with a linear geometry have been published to date. Herein, we describe the mol­ecular and crystal structures of the title compound, which can act as a potential multidentate ligand to various transition-metal ions. The mol­ecular packing of the title compound was further examined with the aid of a Hirshfeld surface analysis.
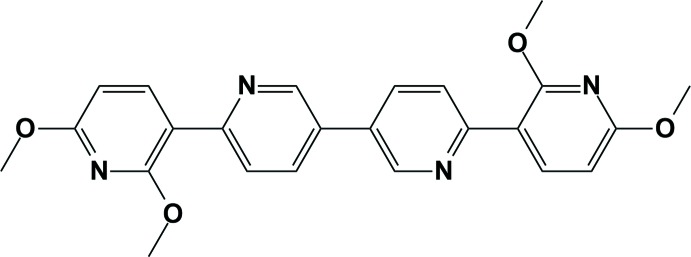


## Structural commentary   

**Figure 1:**
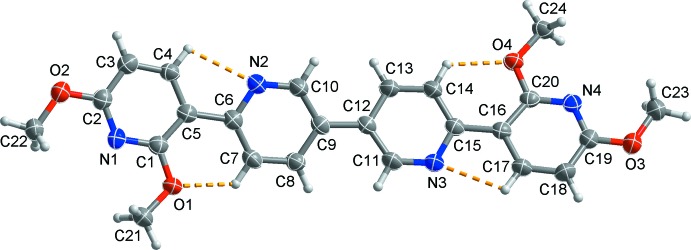
A view of the mol­ecular structure of the title compound, showing the atom-numbering scheme. Displacement ellipsoids are drawn at the 50% probability level. The intra­molecular C—H⋯O/N contacts are shown as yellow dashed lines.

The mol­ecular structure of the title compound is shown in Fig. 1 ▸. Within the mol­ecule, short intra­molecular C—H⋯O and C—H⋯N contacts (Table 1 ▸) enclose *S*(6) and *S*(5) rings, respectively, and may contribute to the planarity between outer and inner pyridine rings. The dihedral angles between the outer and inner pyridine rings are 12.51 (8)° (between rings N1/C1–C5 and N2/C6-C10) and 9.67 (9)° (between rings N3/C11–C15 and N4/C16–C20). However the two inner pyridine rings (N2/C6–C10 and N3/C11–C15) are slightly tilted by 20.10 (7)° with respect to each other. This may be due to the steric hindrance between atoms H8 and H11 and between H10 and H13.

## Supra­molecular features   

**Figure 2:**
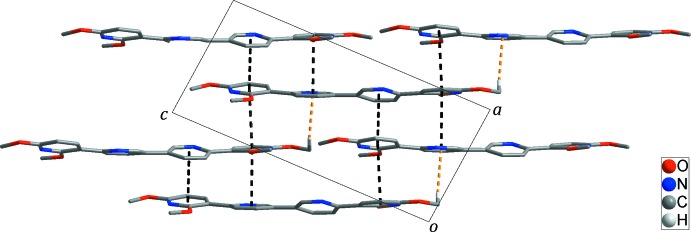
The two-dimensional supra­molecular network formed through π–π stacking inter­actions (black dashed lines) and inter­molecular C—H⋯π inter­actions (yellow dashed lines). For clarity, H atoms not involved in the inter­molecular inter­actions have been omitted.

In the crystal, adjacent mol­ecules are linked by π–π stacking inter­actions between pyridine rings [*Cg*1⋯*Cg*3^iii^ = 3.6600 (10) Å; *Cg*1⋯*Cg*4^ii^ = 3.8249 (10) Å; *Cg*2⋯*Cg*4^iii^ = 3.9270 (10) Å; *Cg*1, *Cg*2, *Cg*3, and *Cg*4 are the centroids of the N1/C1–C5, N2/C6–C10, N3/C11–C15, and N4/C16–C20 rings, respectively; symmetry codes: (ii) *x* + 1, −*y* + 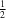, *z* − 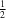, (iii) *x*, −*y* + 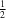, *z* − 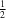], resulting in the formation of a two-dimensional layer structure extending parallel to the *ac* plane, as shown in Fig. 2 ▸. The layer is further stabilized by weak C—H⋯π inter­actions (Table 1 ▸, yellow dashed lines in Fig. 2 ▸) between (meth­yl)H22*C*⋯*Cg*3^i^ [*Cg*3 is the centroid of the N3/C11–C15 ring; symmetry code as in Table 1 ▸]. No inter­actions between the layers are observed.

## Hirshfeld surface analysis   

**Figure 3:**
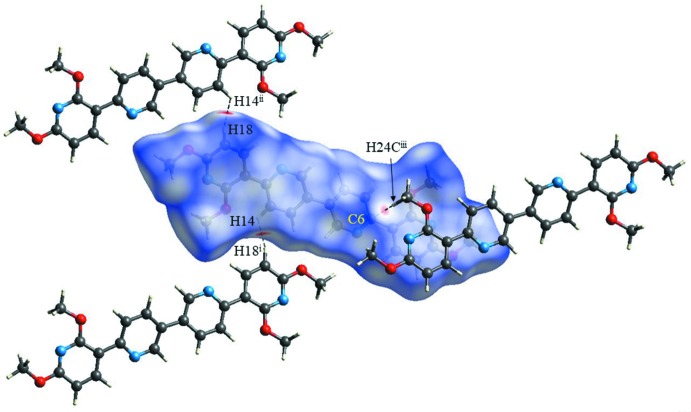
A view of the Hirshfeld surfaced of the title compound mapped over *d*_norm_ showing inter­molecular H⋯H and C⋯H contacts using a fixed colour scale of −0.1883 (red) to 1.2065 (blue) a.u. [Symmetry codes: (i) −*x* + 1, *y* − 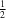, −*z* + 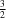; (ii) −*x* + 1, *y* + 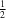, −*z* + 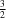; (iii) *x* + 1, −*y* + 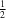, *z* − 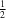.]

Hirshfeld surface analysis was performed using *CrystalExplorer* (Turner *et al.*, 2017 ▸) to qu­antify and visualize the various inter­molecular close contacts in the mol­ecular packing of the title compound. The Hirshfeld surface shown in Fig. 3 ▸ was calculated using a standard (high) surface resolution with the three-dimensional *d*_norm_ surface mapped over a fixed colour scale of −0.1883 (red) to 1.2065 (blue) a.u.. In Fig. 3 ▸, except for three light-red spots, the overall surface mapped over *d*_norm_ is covered by white and blue colours, indicating that the distances between the contact atoms in inter­molecular contacts are nearly the same as the sum of their van der Waals radii or longer. The light-red spots on the surface indicate the closest inter­molecular H⋯H and C⋯H contacts [H14⋯H18(−*x* + 1, *y* − 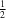, −*z* + 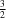) = 2.19 Å, C6⋯H24*C*(*x* + 1, −*y* + 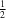, *z* − 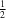) = 2.78 Å.

**Figure 4:**
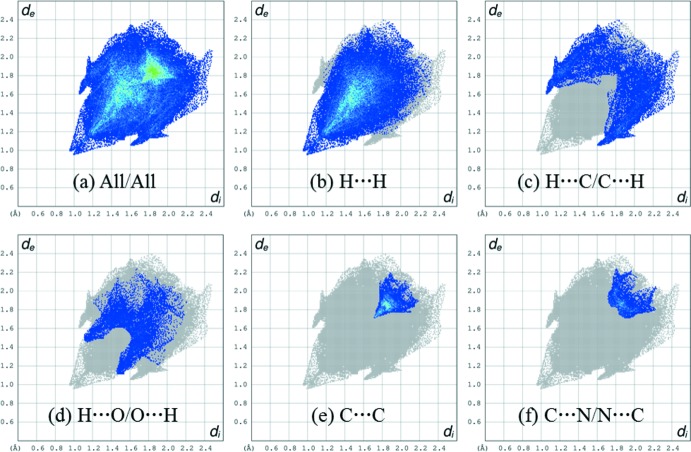
(*a*) The full two-dimensional fingerprint plot for the title compound and those delineated into (*b*) H⋯H, (*c*) H⋯C/C⋯H, (*d*) H···O/O⋯H, (*e*) C⋯C, and (*f*) C⋯N/N⋯C contacts. The *d*_i_ and *d*_e_ values are the closest inter­nal and external distances (in Å) from given points on the Hirshfeld surface contacts.

The overall two-dimensional fingerprint plot and those delineated into H⋯H, H⋯C/C⋯H, H⋯O/O⋯H, C⋯C, and C⋯N/N⋯C contacts are shown in Fig. 4 ▸ *a*–*f*, respectively. The most widely scattered points in the fingerprint plot are related to H⋯H contacts, Fig. 4 ▸ *b*, which make a 52.9% contribution to the Hirshfeld surface. The second largest contribution (17.3%) is by H⋯C/C⋯H contacts (Fig. 4 ▸ *c*). The H⋯O/O⋯H (9.4%), C⋯C (6.4%), C⋯N/N⋯C (5.4%), H⋯N/N⋯H (5.0%), and C⋯O/O⋯C (2.2%) contacts also make significant contributions to the Hirshfeld surface while the N⋯O/O⋯N (0.7%), O⋯O (0.7%), and N⋯N (0.1%) contacts have a negligible influence on the mol­ecular packing.

## Database survey   

Although a search of the Cambridge Structural Database (CSD Version 5.40, last update Feb 2019; Groom *et al.*, 2016 ▸) for 3,2′:5′,3′′:6′′,3′′′-quaterpyridine, which is the title compound without the meth­oxy substituents, and 4,2′:5′,3′′:6′′,4′′′-quaterpyridine gave no hits, that for 2,2′:5′,3′′:6′′,2′′′-quaterpyridine gave ten hits. One (CIHJUB: Luis *et al.*, 2018 ▸) is 2,2′:5′,3′′:6′′,2′′′-quaterpyridine and eight are Ag^I^ (GIWKAY: Baxter *et al.*, 1999 ▸), Cu^I^ (WAHKOF: Baxter *et al.*, 1993 ▸), Ru^II^ (TOMROD: Glasson *et al.*, 2008*a* ▸), or Fe^II^ [(OMAMEV: Glasson *et al.*, 2011**a* ▸;* RIXYON, RIXZAA and RIXYUT: Glasson *et al.*, 2008*b* ▸) complexes involving the 2,2′:5′,3′′:6′′,2′′′-quaterpyridine ligand with methyl substituents. The remaining one (REHVAB: Baxter *et al.*, 1997 ▸) is a Cu^I^ complex involving the ligand 2,2′:5′,3′′:6′′,2′′′-quaterpyridine with phenyl substituents.

## Synthesis and crystallization   

**Figure 5:**
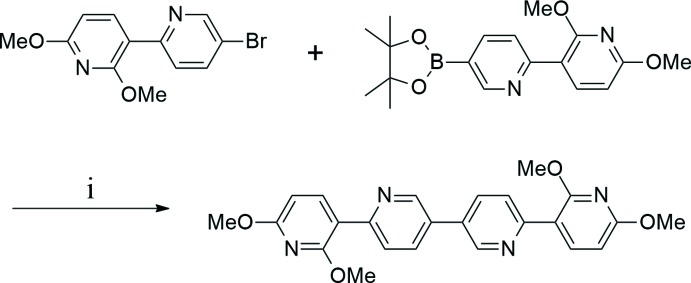
Synthetic routes and reagents to obtain the title compound: (i) Pd(PPh_3_)_4_ (5 mol%), K_3_PO_4_ (6 eq), THF/H_2_O, 373 K, 24 h.

All experiments were performed under a dry N_2_ atmosphere using standard Schlenk techniques. All solvents were freshly distilled over appropriate drying reagents prior to use. All starting materials were purchased commercially and used without further purification. The ^1^H NMR spectrum was recorded on a JEOL 400 MHz spectrometer. The two starting materials, 5-bromo-2′,6′-dimeth­oxy-2,3′-bi­pyridine and 2′,6′-dimeth­oxy-5-(4,4,5,5,-tetra­methyl-1,3,2-dioxaborolan-2-yl)-2,3′-bi­pyridine were synthesized according to a slight modification of the previous synthetic methodology reported by our group (Zaen *et al.*, 2019 ▸). Details of the synthetic procedures and reagents are presented in Fig. 5 ▸.

To a 100 ml Schlenk flask were added 5-bromo-2′,6′-dimeth­oxy-2,3′-bi­pyridine (0.46 g, 1.55 mmol), 2′,6′-dimeth­oxy-5-(4,4,5,5,-tetra­methyl-1,3,2-dioxaborolan-2-yl)-2,3′-bi­pyridine (0.64 g, 1.86 mmol), Pd(PPh_3_)_4_ (0.09 g, 0.08 mmol), and K_3_PO_4_ (2.13 g, 9.28 mmol). The flask was evacuated and back-filled with nitro­gen and THF/H_2_O (12 ml/9.8 ml) was added under an N_2_ atmosphere, and the reaction mixture was stirred at 373 K under nitro­gen for 24 h. After cooling to room temperature, the mixture was poured into 100 ml of water and extracted with ethyl acetate (50 ml × 3). The organic layers were combined and then dried with anhydrous MgSO_4_ and concentrated under reduced pressure. Purification by column chromatography (ethyl­acetate:hexane 1:1, *v*/*v*) afford the desired product as a yellow solid (0.33 g, 50%). Pale-yellow crystals were obtained by slow evaporation of a di­chloro­methane/hexane solution of the title compound. ^1^H NMR (400 MHz, CDCl_3_) δ 8.91 (*dd*, *J* = 2.0 Hz, 2H), 8.32 (*d*, *J* = 8.4 Hz, 2H), 8.10 (*d*, *J* = 7.6 Hz, 2H), 7.93 (*dd*, *J* = 8.4, 2.4 Hz, 2H), 6.47 (*d*, *J* = 8.0 Hz, 2H), 6.47 (*d*, *J* = 8.0 Hz, 2H), 4.06 (*s*, 3H), 3.99 (*s*, 3H); ^13^C NMR(100 MHz, CDCl_3_) δ 163.3, 160.2, 153.8, 147.5, 142.2, 134.2, 130.9, 123.9, 113.8, 102.2, 53.8, 53.6. Analysis calculated for C_24_H_22_N_4_O_4_: C 66.97, H 5.15, N 13.02%; found: C 66.93, H 5.12, N 13.06%.

## Refinement   

Crystal data, data collection and structure refinement details are summarized in Table 2 ▸. All H atoms were positioned geometrically and refined using a riding model: C—H = 0.94–0.97 Å with *U*_iso_(H) = 1.5*U*_eq_(C-meth­yl) and 1.2*U*_eq_(C) for other H atoms.

## Supplementary Material

Crystal structure: contains datablock(s) I, New_Global_Publ_Block. DOI: 10.1107/S205698901901274X/su5516sup1.cif

Structure factors: contains datablock(s) I. DOI: 10.1107/S205698901901274X/su5516Isup2.hkl

Supporting information file. DOI: 10.1107/S205698901901274X/su5516Isup3.cml

CCDC reference: 1953451

Additional supporting information:  crystallographic information; 3D view; checkCIF report

## Figures and Tables

**Table 1:** Hydrogen-bond geometry (Å, °) *Cg*3 is the centroid of the N3/C11–C15 ring.

*D*—H⋯*A*	*D*—H	H⋯*A*	*D*⋯*A*	*D*—H⋯*A*
C7—H7⋯O1	0.94	2.20	2.808 (2)	122
C4—H4⋯N2	0.94	2.41	2.760 (2)	102
C14—H14⋯O4	0.94	2.16	2.808 (2)	125
C17—H17⋯N3	0.94	2.40	2.752 (2)	102
C22—H22*C*⋯*Cg*3^i^	0.97	2.78	3.579 (2)	140

Symmetry code: (i) 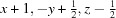.

**Table 2:** Experimental details

Crystal data	
Chemical formula	C_24_H_22_N_4_O_4_
*M*_r_	430.45
Crystal system, space group	Monoclinic, *P*2_1_/*c*
Temperature (K)	223
*a*, *b*, *c* (Å)	7.9556 (6), 14.8583 (11), 17.3362 (12)
β (°)	95.556 (4)
*V* (Å^3^)	2039.6 (3)
*Z*	4
Radiation type	Mo *K*α
μ (mm^−1^)	0.10
Crystal size (mm)	0.25 × 0.24 × 0.07

Data collection	
Diffractometer	Bruker APEXII CCD
Absorption correction	Multi-scan (*SADABS*; Bruker, 2014 ▸)
*T*_min_, *T*_max_	0.673, 0.746
No. of measured, independent and observed [*I* > 2σ(*I*)] reflections	19298, 5090, 3739
*R*_int_	0.030
(sin θ/λ)_max_ (Å^−1^)	0.668

Refinement	
*R*[*F* ^2^ > 2σ(*F* ^2^)], *wR*(*F* ^2^), *S*	0.054, 0.160, 1.04
No. of reflections	5090
No. of parameters	289
H-atom treatment	H-atom parameters constrained
Δρ_max_, Δρ_min_ (e Å^−3^)	0.52, −0.24

Computer programs: *APEX2* and *SAINT* (Bruker, 2014 ▸), *SHELXS97* and *SHELXTL* (Sheldrick, 2008 ▸), *SHELXL2014/7* (Sheldrick, 2015 ▸), *DIAMOND* (Brandenburg, 2010 ▸) and *publCIF* (Westrip, 2010 ▸).

## supplementary crystallographic information

### Crystal data

**Table:**

C_24_H_22_N_4_O_4_	*F*(000) = 904
*M**_r_* = 430.45	*D*_x_ = 1.402 Mg m^−^^3^
Monoclinic, *P*2_1_/*c*	Mo *K*α radiation, λ = 0.71073 Å
*a* = 7.9556 (6) Å	Cell parameters from 5328 reflections
*b* = 14.8583 (11) Å	θ = 2.6–27.8°
*c* = 17.3362 (12) Å	µ = 0.10 mm^−^^1^
β = 95.556 (4)°	*T* = 223 K
*V* = 2039.6 (3) Å^3^	Plate, yellow
*Z* = 4	0.25 × 0.24 × 0.07 mm

### Data collection

**Table:**

Bruker APEXII CCD diffractometer	3739 reflections with *I* > 2σ(*I*)
φ and ω scans	*R*_int_ = 0.030
Absorption correction: multi-scan (SADABS; Bruker, 2014)	θ_max_ = 28.4°, θ_min_ = 1.8°
*T*_min_ = 0.673, *T*_max_ = 0.746	*h* = −10→10
19298 measured reflections	*k* = −17→19
5090 independent reflections	*l* = −23→23

### Refinement

**Table:**

Refinement on *F*^2^	Primary atom site location: structure-invariant direct methods
Least-squares matrix: full	Secondary atom site location: difference Fourier map
*R*[*F*^2^ > 2σ(*F*^2^)] = 0.054	Hydrogen site location: inferred from neighbouring sites
*wR*(*F*^2^) = 0.160	H-atom parameters constrained
*S* = 1.04	*w* = 1/[σ^2^(*F*_o_^2^) + (0.0773*P*)^2^ + 0.5757*P*] where *P* = (*F*_o_^2^ + 2*F*_c_^2^)/3
5090 reflections	(Δ/σ)_max_ < 0.001
289 parameters	Δρ_max_ = 0.52 e Å^−^^3^
0 restraints	Δρ_min_ = −0.24 e Å^−^^3^

### Special details

**Table:**

Geometry. All esds (except the esd in the dihedral angle between two l.s. planes) are estimated using the full covariance matrix. The cell esds are taken into account individually in the estimation of esds in distances, angles and torsion angles; correlations between esds in cell parameters are only used when they are defined by crystal symmetry. An approximate (isotropic) treatment of cell esds is used for estimating esds involving l.s. planes.

### Fractional atomic coordinates and isotropic or equivalent isotropic displacement parameters (Å^2^)

**Table:**

	*x*	*y*	*z*	*U*_iso_*/*U*_eq_	
O1	0.95531 (17)	0.30630 (8)	0.18517 (7)	0.0449 (3)	
O2	1.11979 (18)	0.05085 (9)	0.06420 (7)	0.0518 (3)	
O3	0.3179 (2)	0.49944 (9)	0.92984 (8)	0.0598 (4)	
O4	0.34148 (15)	0.25399 (7)	0.77125 (7)	0.0414 (3)	
N1	1.03508 (18)	0.17731 (10)	0.12555 (8)	0.0393 (3)	
N2	0.8189 (2)	0.15075 (10)	0.37266 (9)	0.0473 (4)	
N3	0.5594 (2)	0.41616 (10)	0.60438 (8)	0.0446 (4)	
N4	0.33254 (18)	0.37725 (10)	0.84952 (8)	0.0394 (3)	
C1	0.9663 (2)	0.21499 (11)	0.18490 (9)	0.0384 (4)	
C2	1.0519 (2)	0.08936 (12)	0.12445 (10)	0.0417 (4)	
C3	1.0021 (2)	0.03287 (13)	0.18274 (11)	0.0464 (4)	
H3	1.0169	−0.0299	0.1812	0.056*	
C4	0.9309 (2)	0.07415 (11)	0.24195 (10)	0.0391 (4)	
H4	0.8953	0.0382	0.2819	0.047*	
C5	0.9084 (2)	0.16701 (12)	0.24608 (9)	0.0385 (4)	
C6	0.8322 (2)	0.20736 (12)	0.31288 (9)	0.0378 (4)	
C7	0.7761 (2)	0.29583 (12)	0.31670 (10)	0.0426 (4)	
H7	0.7840	0.3347	0.2744	0.051*	
C8	0.7093 (2)	0.32678 (12)	0.38209 (10)	0.0416 (4)	
H8	0.6730	0.3868	0.3845	0.050*	
C9	0.6954 (2)	0.26934 (11)	0.44454 (9)	0.0362 (4)	
C10	0.7519 (2)	0.18211 (13)	0.43460 (10)	0.0461 (4)	
H10	0.7420	0.1415	0.4755	0.055*	
C11	0.6163 (2)	0.38702 (12)	0.53909 (10)	0.0454 (4)	
H11	0.6530	0.4306	0.5052	0.055*	
C12	0.6258 (2)	0.29739 (11)	0.51692 (9)	0.0352 (3)	
C13	0.5675 (2)	0.23564 (12)	0.56830 (10)	0.0443 (4)	
H13	0.5692	0.1739	0.5567	0.053*	
C14	0.5075 (2)	0.26401 (12)	0.63572 (10)	0.0441 (4)	
H14	0.4672	0.2217	0.6698	0.053*	
C15	0.5059 (2)	0.35488 (11)	0.65387 (9)	0.0350 (3)	
C16	0.4526 (2)	0.39106 (11)	0.72707 (9)	0.0363 (4)	
C17	0.4828 (3)	0.48144 (12)	0.74535 (11)	0.0464 (4)	
H17	0.5351	0.5179	0.7103	0.056*	
C18	0.4387 (3)	0.51856 (13)	0.81250 (12)	0.0564 (5)	
H18	0.4592	0.5796	0.8241	0.068*	
C19	0.3625 (2)	0.46282 (12)	0.86309 (10)	0.0455 (4)	
C20	0.3758 (2)	0.34235 (11)	0.78363 (9)	0.0350 (4)	
C21	1.0326 (3)	0.35294 (14)	0.12610 (11)	0.0546 (5)	
H21A	1.0169	0.4172	0.1320	0.082*	
H21B	1.1524	0.3393	0.1304	0.082*	
H21C	0.9813	0.3340	0.0757	0.082*	
C22	1.1533 (3)	0.10987 (14)	0.00148 (10)	0.0497 (5)	
H22A	1.2017	0.0755	−0.0385	0.075*	
H22B	1.0487	0.1375	−0.0200	0.075*	
H22C	1.2321	0.1563	0.0208	0.075*	
C23	0.2306 (3)	0.44212 (15)	0.97894 (12)	0.0624 (6)	
H23A	0.2054	0.4753	1.0246	0.094*	
H23B	0.1261	0.4215	0.9510	0.094*	
H23C	0.3010	0.3907	0.9945	0.094*	
C24	0.2705 (2)	0.20561 (12)	0.83165 (11)	0.0444 (4)	
H24A	0.2518	0.1435	0.8159	0.067*	
H24B	0.3477	0.2077	0.8785	0.067*	
H24C	0.1638	0.2328	0.8415	0.067*	

### Atomic displacement parameters (Å^2^)

**Table:**

	*U*^11^	*U*^22^	*U*^33^	*U*^12^	*U*^13^	*U*^23^
O1	0.0596 (8)	0.0332 (6)	0.0437 (7)	−0.0006 (5)	0.0152 (6)	0.0045 (5)
O2	0.0633 (9)	0.0451 (7)	0.0486 (7)	0.0003 (6)	0.0136 (6)	−0.0066 (6)
O3	0.0871 (11)	0.0448 (8)	0.0512 (8)	−0.0013 (7)	0.0261 (7)	−0.0053 (6)
O4	0.0477 (7)	0.0327 (6)	0.0458 (7)	−0.0051 (5)	0.0141 (5)	0.0031 (5)
N1	0.0379 (8)	0.0435 (8)	0.0362 (7)	0.0006 (6)	0.0031 (6)	−0.0048 (6)
N2	0.0558 (10)	0.0450 (9)	0.0424 (8)	0.0017 (7)	0.0119 (7)	0.0060 (6)
N3	0.0571 (9)	0.0351 (8)	0.0430 (8)	−0.0065 (7)	0.0125 (7)	0.0027 (6)
N4	0.0404 (8)	0.0381 (8)	0.0402 (7)	0.0044 (6)	0.0060 (6)	0.0037 (6)
C1	0.0378 (9)	0.0381 (9)	0.0388 (8)	−0.0010 (7)	0.0008 (7)	0.0019 (7)
C2	0.0391 (9)	0.0436 (10)	0.0422 (9)	−0.0023 (7)	0.0028 (7)	−0.0034 (7)
C3	0.0482 (10)	0.0397 (9)	0.0516 (10)	0.0002 (8)	0.0056 (8)	−0.0033 (8)
C4	0.0404 (9)	0.0364 (9)	0.0409 (9)	−0.0018 (7)	0.0062 (7)	0.0055 (7)
C5	0.0361 (9)	0.0390 (9)	0.0399 (9)	−0.0020 (7)	0.0016 (7)	0.0026 (7)
C6	0.0319 (8)	0.0440 (9)	0.0371 (8)	−0.0014 (7)	0.0021 (6)	0.0030 (7)
C7	0.0449 (10)	0.0464 (10)	0.0374 (8)	−0.0006 (8)	0.0084 (7)	0.0101 (7)
C8	0.0433 (10)	0.0393 (9)	0.0433 (9)	0.0033 (7)	0.0100 (7)	0.0065 (7)
C9	0.0310 (8)	0.0426 (9)	0.0352 (8)	−0.0032 (7)	0.0036 (6)	0.0041 (7)
C10	0.0547 (11)	0.0435 (10)	0.0412 (9)	0.0031 (8)	0.0111 (8)	0.0085 (7)
C11	0.0563 (11)	0.0381 (9)	0.0437 (9)	−0.0079 (8)	0.0140 (8)	0.0069 (7)
C12	0.0313 (8)	0.0390 (9)	0.0353 (8)	−0.0020 (6)	0.0025 (6)	0.0041 (6)
C13	0.0553 (11)	0.0335 (9)	0.0464 (9)	−0.0007 (8)	0.0159 (8)	0.0032 (7)
C14	0.0549 (11)	0.0367 (9)	0.0429 (9)	0.0002 (8)	0.0158 (8)	0.0076 (7)
C15	0.0322 (8)	0.0363 (8)	0.0364 (8)	−0.0005 (6)	0.0029 (6)	0.0043 (6)
C16	0.0364 (8)	0.0340 (8)	0.0383 (8)	0.0008 (7)	0.0029 (7)	0.0048 (6)
C17	0.0615 (12)	0.0349 (9)	0.0445 (9)	−0.0049 (8)	0.0138 (8)	0.0045 (7)
C18	0.0851 (15)	0.0332 (9)	0.0532 (11)	−0.0053 (9)	0.0175 (10)	−0.0028 (8)
C19	0.0563 (11)	0.0398 (10)	0.0416 (9)	0.0041 (8)	0.0103 (8)	0.0008 (7)
C20	0.0318 (8)	0.0328 (8)	0.0402 (8)	0.0032 (6)	0.0023 (6)	0.0038 (6)
C21	0.0740 (14)	0.0475 (11)	0.0449 (10)	−0.0004 (9)	0.0183 (9)	0.0089 (8)
C22	0.0585 (12)	0.0524 (11)	0.0399 (9)	−0.0001 (9)	0.0129 (8)	−0.0029 (8)
C23	0.0874 (16)	0.0519 (12)	0.0522 (11)	0.0038 (11)	0.0292 (11)	0.0029 (9)
C24	0.0453 (10)	0.0396 (9)	0.0500 (10)	−0.0034 (8)	0.0134 (8)	0.0097 (7)

### Geometric parameters (Å, º)

**Table:**

O1—C1	1.359 (2)	C9—C12	1.480 (2)
O1—C21	1.425 (2)	C10—H10	0.9400
O2—C2	1.349 (2)	C11—C12	1.390 (2)
O2—C22	1.442 (2)	C11—H11	0.9400
O3—C19	1.357 (2)	C12—C13	1.389 (2)
O3—C23	1.431 (2)	C13—C14	1.371 (2)
O4—C20	1.3538 (19)	C13—H13	0.9400
O4—C24	1.4308 (19)	C14—C15	1.387 (2)
N1—C2	1.314 (2)	C14—H14	0.9400
N1—C1	1.334 (2)	C15—C16	1.478 (2)
N2—C10	1.328 (2)	C16—C17	1.395 (2)
N2—C6	1.347 (2)	C16—C20	1.405 (2)
N3—C11	1.331 (2)	C17—C18	1.364 (3)
N3—C15	1.348 (2)	C17—H17	0.9400
N4—C19	1.311 (2)	C18—C19	1.388 (3)
N4—C20	1.330 (2)	C18—H18	0.9400
C1—C5	1.393 (2)	C21—H21A	0.9700
C2—C3	1.400 (3)	C21—H21B	0.9700
C3—C4	1.365 (2)	C21—H21C	0.9700
C3—H3	0.9400	C22—H22A	0.9700
C4—C5	1.394 (2)	C22—H22B	0.9700
C4—H4	0.9400	C22—H22C	0.9700
C5—C6	1.485 (2)	C23—H23A	0.9700
C6—C7	1.392 (3)	C23—H23B	0.9700
C7—C8	1.377 (2)	C23—H23C	0.9700
C7—H7	0.9400	C24—H24A	0.9700
C8—C9	1.391 (2)	C24—H24B	0.9700
C8—H8	0.9400	C24—H24C	0.9700
C9—C10	1.388 (3)		
			
C1—O1—C21	116.78 (14)	C12—C13—H13	119.7
C2—O2—C22	116.31 (14)	C13—C14—C15	120.29 (16)
C19—O3—C23	116.79 (15)	C13—C14—H14	119.9
C20—O4—C24	117.33 (13)	C15—C14—H14	119.9
C2—N1—C1	118.58 (15)	N3—C15—C14	120.19 (15)
C10—N2—C6	118.06 (15)	N3—C15—C16	115.81 (14)
C11—N3—C15	118.40 (15)	C14—C15—C16	123.97 (15)
C19—N4—C20	118.35 (15)	C17—C16—C20	114.46 (15)
N1—C1—O1	116.91 (15)	C17—C16—C15	119.20 (15)
N1—C1—C5	124.26 (16)	C20—C16—C15	126.31 (15)
O1—C1—C5	118.82 (15)	C18—C17—C16	122.02 (17)
N1—C2—O2	118.82 (16)	C18—C17—H17	119.0
N1—C2—C3	123.33 (16)	C16—C17—H17	119.0
O2—C2—C3	117.84 (16)	C17—C18—C19	117.52 (17)
C4—C3—C2	116.12 (16)	C17—C18—H18	121.2
C4—C3—H3	121.9	C19—C18—H18	121.2
C2—C3—H3	121.9	N4—C19—O3	118.96 (16)
C3—C4—C5	123.24 (16)	N4—C19—C18	123.26 (17)
C3—C4—H4	118.4	O3—C19—C18	117.77 (17)
C5—C4—H4	118.4	N4—C20—O4	116.70 (14)
C1—C5—C4	114.45 (15)	N4—C20—C16	124.38 (15)
C1—C5—C6	125.27 (16)	O4—C20—C16	118.91 (14)
C4—C5—C6	120.26 (15)	O1—C21—H21A	109.5
N2—C6—C7	120.26 (16)	O1—C21—H21B	109.5
N2—C6—C5	114.61 (15)	H21A—C21—H21B	109.5
C7—C6—C5	125.12 (15)	O1—C21—H21C	109.5
C8—C7—C6	120.37 (16)	H21A—C21—H21C	109.5
C8—C7—H7	119.8	H21B—C21—H21C	109.5
C6—C7—H7	119.8	O2—C22—H22A	109.5
C7—C8—C9	120.13 (16)	O2—C22—H22B	109.5
C7—C8—H8	119.9	H22A—C22—H22B	109.5
C9—C8—H8	119.9	O2—C22—H22C	109.5
C10—C9—C8	115.14 (15)	H22A—C22—H22C	109.5
C10—C9—C12	121.38 (15)	H22B—C22—H22C	109.5
C8—C9—C12	123.48 (15)	O3—C23—H23A	109.5
N2—C10—C9	126.02 (16)	O3—C23—H23B	109.5
N2—C10—H10	117.0	H23A—C23—H23B	109.5
C9—C10—H10	117.0	O3—C23—H23C	109.5
N3—C11—C12	125.36 (16)	H23A—C23—H23C	109.5
N3—C11—H11	117.3	H23B—C23—H23C	109.5
C12—C11—H11	117.3	O4—C24—H24A	109.5
C13—C12—C11	115.14 (15)	O4—C24—H24B	109.5
C13—C12—C9	122.22 (15)	H24A—C24—H24B	109.5
C11—C12—C9	122.63 (15)	O4—C24—H24C	109.5
C14—C13—C12	120.59 (16)	H24A—C24—H24C	109.5
C14—C13—H13	119.7	H24B—C24—H24C	109.5
			
C2—N1—C1—O1	178.19 (15)	N3—C11—C12—C9	−178.07 (17)
C2—N1—C1—C5	−0.8 (2)	C10—C9—C12—C13	−19.2 (3)
C21—O1—C1—N1	−5.8 (2)	C8—C9—C12—C13	160.80 (17)
C21—O1—C1—C5	173.33 (16)	C10—C9—C12—C11	159.83 (17)
C1—N1—C2—O2	179.34 (15)	C8—C9—C12—C11	−20.2 (3)
C1—N1—C2—C3	−0.4 (3)	C11—C12—C13—C14	−0.6 (3)
C22—O2—C2—N1	−6.0 (2)	C9—C12—C13—C14	178.47 (17)
C22—O2—C2—C3	173.76 (16)	C12—C13—C14—C15	−0.7 (3)
N1—C2—C3—C4	1.0 (3)	C11—N3—C15—C14	−1.3 (3)
O2—C2—C3—C4	−178.75 (15)	C11—N3—C15—C16	176.97 (15)
C2—C3—C4—C5	−0.4 (3)	C13—C14—C15—N3	1.7 (3)
N1—C1—C5—C4	1.3 (2)	C13—C14—C15—C16	−176.46 (17)
O1—C1—C5—C4	−177.71 (15)	N3—C15—C16—C17	−9.1 (2)
N1—C1—C5—C6	179.71 (15)	C14—C15—C16—C17	169.13 (18)
O1—C1—C5—C6	0.7 (2)	N3—C15—C16—C20	172.75 (15)
C3—C4—C5—C1	−0.6 (2)	C14—C15—C16—C20	−9.0 (3)
C3—C4—C5—C6	−179.12 (16)	C20—C16—C17—C18	−0.7 (3)
C10—N2—C6—C7	−0.3 (3)	C15—C16—C17—C18	−179.08 (18)
C10—N2—C6—C5	179.94 (16)	C16—C17—C18—C19	0.3 (3)
C1—C5—C6—N2	−166.43 (16)	C20—N4—C19—O3	179.54 (16)
C4—C5—C6—N2	11.9 (2)	C20—N4—C19—C18	−0.7 (3)
C1—C5—C6—C7	13.8 (3)	C23—O3—C19—N4	−3.8 (3)
C4—C5—C6—C7	−167.89 (17)	C23—O3—C19—C18	176.41 (19)
N2—C6—C7—C8	1.1 (3)	C17—C18—C19—N4	0.4 (3)
C5—C6—C7—C8	−179.12 (16)	C17—C18—C19—O3	−179.80 (19)
C6—C7—C8—C9	−0.8 (3)	C19—N4—C20—O4	−179.38 (15)
C7—C8—C9—C10	−0.3 (2)	C19—N4—C20—C16	0.2 (2)
C7—C8—C9—C12	179.71 (16)	C24—O4—C20—N4	−3.2 (2)
C6—N2—C10—C9	−1.0 (3)	C24—O4—C20—C16	177.18 (15)
C8—C9—C10—N2	1.2 (3)	C17—C16—C20—N4	0.5 (2)
C12—C9—C10—N2	−178.79 (17)	C15—C16—C20—N4	178.68 (15)
C15—N3—C11—C12	0.0 (3)	C17—C16—C20—O4	−179.93 (15)
N3—C11—C12—C13	1.0 (3)	C15—C16—C20—O4	−1.7 (2)

### Hydrogen-bond geometry (Å, º)

*Cg*3 is the centroid of the N3/C11–C15 ring.

**Table:**

*D*—H···*A*	*D*—H	H···*A*	*D*···*A*	*D*—H···*A*
C7—H7···O1	0.94	2.20	2.808 (2)	122
C4—H4···N2	0.94	2.41	2.760 (2)	102
C14—H14···O4	0.94	2.16	2.808 (2)	125
C17—H17···N3	0.94	2.40	2.752 (2)	102
C22—H22*C*···*Cg*3^i^	0.97	2.78	3.579 (2)	140

Symmetry code: (i) *x*+1, −*y*+1/2, *z*−1/2.
